# MetazSecKB: the human and animal secretome and subcellular proteome knowledgebase

**DOI:** 10.1093/database/bav077

**Published:** 2015-08-08

**Authors:** John Meinken, Gary Walker, Chester R. Cooper, Xiang Jia Min

**Affiliations:** ^1^Department of Computer Science and Information Systems,; ^2^Center for Applied Chemical Biology and; ^3^Department of Biological Sciences, Youngstown State University, Youngstown, OH 44555, USA

## Abstract

The subcellular location of a protein is a key factor in determining the molecular function of the protein in an organism. MetazSecKB is a secretome and subcellular proteome knowledgebase specifically designed for metazoan, i.e. human and animals. The protein sequence data, consisting of over 4 million entries with 121 species having a complete proteome, were retrieved from UniProtKB. Protein subcellular locations including secreted and 15 other subcellular locations were assigned based on either curated experimental evidence or prediction using seven computational tools. The protein or subcellular proteome data can be searched and downloaded using several different types of identifiers, gene name or keyword(s), and species. BLAST search and community annotation of subcellular locations are also supported. Our primary analysis revealed that the proteome sizes, secretome sizes and other subcellular proteome sizes vary tremendously in different animal species. The proportions of secretomes vary from 3 to 22% (average 8%) in metazoa species*.* The proportions of other major subcellular proteomes ranged approximately 21–43% (average 31%) in cytoplasm, 20–37% (average 30%) in nucleus, 3–19% (average 12%) as plasma membrane proteins and 3–9% (average 6%) in mitochondria. We also compared the protein families in secretomes of different primates. The Gene Ontology and protein family domain analysis of human secreted proteins revealed that these proteins play important roles in regulation of human structure development, signal transduction, immune systems and many other biological processes.

**Database URL:** http://proteomics.ysu.edu/secretomes/animal/index.php

## Introduction

Secreted proteins play important roles in the development of multicellular organisms, serving as signal molecules, extracellular enzymes and structural matrix. The first sequenced protein, human insulin, was actually a secreted protein. Human secreted proteins have potential to be used as biomarkers for the diagnosis of diseases (1). The term ‘secretome’ was first used by Tjalsma *et al*. (2) to include all proteins that are synthesized and processed by the secretary pathway and proteins located in the secretion machinery. However, the term recently was limited to include only the set of secreted or extracellular proteins in a species (3, 4). The secretome plays a central role in creating an extracellular environment that allows for physiological coordination and maintaining the homeostatic conditions that support cellular life and thus the organism.

Because of biomedical importance, secretome identification and analysis have been carried out in a number of human and animal cells or tissues including human arterial smooth muscle cells (5), human oligodendrocytes (6), human mesenchymal stem cells (7), human and mouse preimplantation embryos (8), primary human adipocytes during insulin resistance (9), rat adipose tissues (10), 23 cancer cell lines (11), and different types of human primary cell cultures and human body fluids including plasma, cerebrospinal fluid and urine (12). In addition to experimental characterization of human secretomes in various cell types, proteome-wide computational prediction of secretomes has been performed in mouse (13), human, pufferfish, pigs, and zebrafish (14, 15). A secreted protein database was developed for human, rat and mouse, but unfortunately this database has not been updated since 2006 (http://spd.cbi.pku.edu.cn/) (16), and another database, LOCATE, describing the membrane organization and subcellular location including secreted proteins was developed for mouse and human only (http://locate.imb.uq.edu.au/) (17). However, as the complete genome sequencing projects have generated many complete proteome data in animal species, a database having information for computational prediction and curated information of secretomes and other subcellular proteomes in these species would provide a useful resource for both searching an individual protein subcellular location and performing proteome-wide comparative analysis.

In this work, we describe MetazSecKB, the Metazoan, i.e. human and animals, Secretome and Subcellular Proteome Knowledgebase. MetazSecKB is constructed with all available human and animal protein sequences by combining curated subcellular information and predicted information, with a well tested computational protocol, on secretomes and other subcellular proteomes of 15 subcellular locations. This knowledgebase is expected to serve as a central portal for providing information on metazoan protein subcellular locations for biological and medical researchers interested in protein biology.

## Data collection and database implementation

### Data collection

The protein sequences for the kingdom Animalia, also called Metazoa, were retrieved from the UniProtKB/Swiss-Prot dataset and the UniProtKB/TrEMBL dataset (release 2014_01) (http://www.uniprot.org/downloads). The UniProtKB/Swiss-Prot dataset contains manually annotated and reviewed protein sequences with information extracted from literature of experimental results and curator-evaluated computational analysis (18). The UniProtKB/TrEMBL dataset contains computationally analysed protein sequences. The combined metazoan dataset consisted of a total of 4 080 818 protein entries with 103 088 and 3 977 730 entries from the UniProtKB/Swiss-Prot dataset and the UniProtKB/TrEMBL dataset, respectively. The identifier mapping data including UniProt accession number (AC), UniProt ID, RefSeq accession number and gi number were retrieved from the UniProt ID mapping data file.

### Protein subcellular localization prediction

We have previously evaluated several computational tools for predicting classic secreted proteins, i.e. proteins having a secretory signal peptide at the N-terminus (19) (Min 2010). These tools were chosen because they have relatively high prediction accuracy and are available as standalone tools for local processing of large datasets. The protein sequences were processed using the following programs: SignalP (version 3.0 and 4.0) (20, 21), Phobius (22), WoLF PSORT (23) and TargetP (24) for secretory signal peptide and subcellular location prediction. TMHMM (version 2.0) was used to identify proteins having transmembrane domains (25) and Scan-Prosite (called PS-Scan in standalone version) (http://www.expasy.org/tools/scanprosite/) was used to scan endoplasmic reticulum (ER) targeting sequence (Prosite: PS00014) (26, 27). Proteins having one or more membrane domains, but not located within the N-terminus (the first 70 amino acids), were predicted as membrane proteins by TMHMM. The tools mentioned above were installed on a local Linux system for data processing. The commands for running these tools were summarized by Lum and Min (28). Protein sequences predicted to have a signal peptide by SignalP (version 3) were further processed using FragAnchor webserver to identify the glycosylphosphatidyinositol (GPI) anchors (http://navet.ics.hawaii.edu/∼fraganchor/NNHMM/NNHMM.html) (29). These tools have been used for processing fungal and plant protein sequences in construction of FunSecKB (3), FunSecKB2 (4) and PlantSecKB (30). However, based on our previous evaluations, the detailed methods were slightly different for assigning secretomes in different kingdoms of eukaryotes (19).

The metazoan protein subcellular locations are classified into the following categories: secreted proteins, mitochondrial (membrane or non-membrane), ER (membrane or lumen), cytosol (cytoplasm), cytoskeleton, Golgi apparatus (membrane or lumen), nuclear (membrane or non-membrane), vacuolar (membrane or non-membrane), lysosome, peroxisome, plasma membrane, other membrane and GPI-anchored proteins. For assigning a protein subcellular location, the UniProtKB subcellular annotation information was considered prior to using prediction information. For proteins not having annotated subcellular information, their subcellular location assignments are based on computational prediction. In this work, SignalP4 is used to replace SignalP3 as SignalP4 improves the prediction accuracy (21, 31). However, the information generated by SignalP3 was also included as it predicts signal peptide cleavage sites more accurately than SignalP4 (21). The rules for assigning a protein subcellular location are defined below.

#### Secreted protein

Secreted proteins are further divided as curated secreted proteins, highly likely secreted, likely secreted, and weakly likely secreted. Curated secreted proteins are proteins that are annotated and reviewed to be ‘secreted’ or ‘extracellular’ in the subcellular location from the UniProtKB/Swiss-Prot dataset. Four predictors consisting of SignalP4, Phobius, TargetP and WoLF PSORT are used for protein secretory signal peptide or subcellular location prediction (19). The highly likely secreted, likely secreted and weakly likely secreted proteins are proteins that are predicted to be secreted or contain a secretory signal peptide by four and three, two or one of the four tools, respectively. The accuracies for these subcategories of secreted proteins are reported in the section of results. It should be noted that proteins having a transmembrane domain or an ER retention signal were excluded from this set. We recommend that the data for making up a secretome should consist of curated secreted proteins and the predicted highly likely secreted protein dataset. The rational for having subcategories of likely secreted and weakly likely secreted proteins is to provide a means for a user to access these data as some of them may be real secreted proteins.

#### Mitochondrial proteins

A protein predicted as ‘M’ (for mitochondrial) for subcellular location by TargetP and ‘mito’ by WoLF PSORT is classified as a mitochondrial protein. The accuracy is reported in the result. If it is also classified as a membrane protein by TMHMM, then it is further classified as mitochondrial membrane protein.

### ER proteins

ER proteins were predicted using WoLF PSORT and PS-Scan. If they contain one or more transmembrane domains, they are classified as ER membrane proteins. Otherwise, they are classified as ER luminal proteins. Proteins predicted to contain a signal peptide by SignalP 4.0 and an ER target signal (Prosite: PS00014) by PS-Scan often are luminal ER proteins.

#### GPI-anchored proteins

Signal peptide containing proteins that were predicted to have a GPI anchor by FragAnchor were further classified as GPI-anchored proteins. Protein sequences predicted to have a signal peptide and a GPI anchor may attach to the outer leaflet of the plasma membrane or are secreted, thereby becoming components of the extracellular matrix.

#### Proteins in other subcellular locations

Other subcellular locations, including cytoplasm (cytosol), cytoskeleton, Golgi apparatus, lysosome, nucleus, peroxisome, plasma membrane and vacuole, were predicted by WoLF PSORT. For a protein predicted as located in Golgi apparatus, nucleus or vacuole, it was further classified as a membrane protein in that specific subcellular location if it contained one or more transmembrane domain predicted by TMHMM.

### Database implementation

The protein sequence data, species information, subcellular annotation and information predicted from the tools mentioned above were formatted into tab-delimited text files and were stored in a relational database using MySQL hosted in a Linux server. The user interface and modules to access the data were implemented using PHP. BLAST utility and community annotation submission can be accessed from links on the main user interface at http://proteomics.ysu.edu/secretomes/animal/index.php. The supplementary tables and all other data described in the work can be downloaded at http://proteomics.ysu.edu/publication/data/MetazSecKB/.

### Evaluation of prediction accuracies of protein subcellular locations

The prediction tools we employed above were based on our previous evaluation (19, 31, 32). To further evaluate the prediction accuracies of our rule-based methods for each subcellular location in this dataset, we retrieved protein entries having an annotated, unique subcellular location from UniProtKB/Swiss-Prot dataset. Proteins having multiple subcellular locations or labeled as ‘fragment’ or not starting with ‘M’ or having a length < 70 amino acids were excluded. Protein entries having a term including ‘By similarity’, ‘Probable’ or ‘Potential’ in their subcellular location annotation were excluded. The prediction accuracy for each subcellular location was evaluated using prediction sensitivity (Equation 1), specificity (Equation 2) and Matthews Correlation Coefficient (MCC) (Equation 3) (33).

(1) Sensitivity (%) = TP/(TP + FN) × 100

(2) Specificity (%) = TN/(TN + FP) × 100

(3) $$\begin{array}{l} \text{MCC}\left(\%\right)\;=\;(\text{TP}\times \text{TN}-\text{FP}\times \text{FN})\times \text{1}00\;/\\ \left(\left(\text{TP}+\text{FP}\right)\;\left(\text{TP}+\text{FN}\right)\;\left(\text{TN}+\text{FP}\right)\;\left(\text{TN}+\text{FN}\right)\right)\text{1}/\text{2} \end{array}$$

TP is the number of true positives, FN is the number of false negatives, FP is the number of false positives and TN is the number of true negatives. The MCC is used as a measure of the quality of binary (two-class) classifications. It takes into account true and false positives and negatives and is generally regarded as a balanced measure. The MCC returns a value between −1 and +1. A coefficient of +1 represents a perfect prediction, 0 means no better than random prediction, and −1 indicates total disagreement between prediction and observation (33). The dataset contains a total of 18,874 proteins. For each category, the number of actual positives equals TP plus FN and the number of actual negatives equals FP plus TN (Table 1). As both TargetP and WoLF PSORT can predict mitochondrial proteins, we evaluated their prediction accuracy, either used individually or combined, using a dataset consisting of 1870 annotated mitochondrial proteins as positives and 17 004 proteins located in other subcellular locations as negatives.

**Table 1.** Prediction accuracy evaluation of human and animal protein subcellular locations^a^

	TP	FP	TN	FN	Sn (%)	Sp (%)	MCC
(a) Mitochondrial proteins							
TargetP	930	972	16 032	940	49.7	94.3	0.44
WoLF PSORT	920	482	16 522	950	49.2	97.2	0.53
TargetP AND WoLF PSORT	794	262	16 742	1076	42.5	98.5	0.53
TaregetP OR WoLF PSORT	1056	1202	15 802	814	56.5	92.9	0.45
(b) Secreted proteins^b^							
Secreted	5024	276	12 874	700	87.8	97.9	0.88
S + HLS	5350	522	12 628	374	93.5	96.0	0.89
S + HLS + LS	5413	794	12 356	311	94.6	94.0	0.87
S + HLS + LS + WLS	5440	1462	11 688	284	95.0	88.9	0.80
(c) The subcellular locations							
Cytoplasm	1095	1124	15 779	876	55.6	93.4	0.46
Cytoskeleton	218	63	18 020	573	27.6	99.7	0.45
ER	257	187	17 906	524	32.9	99.0	0.42
Golgi	12	21	18 584	257	4.5	99.9	0.12
Lysosome	1	8	18 675	190	0.5	100.0	0.02
Nucleus	2979	893	14 190	812	78.6	94.1	0.72
Peroxisome	4	101	18 653	116	3.3	99.5	0.03
Plasma membrane	2767	647	14 880	580	82.7	95.8	0.78
Vacuole	0	0	18 855	19	0.0	100.0	-

Note: FP, false positives; FN, false negatives; MCC, Matthews correlation coefficient; Sn, sensitivity; Sp, specificity; TP, true positives; TN, true negatives.

^a^The dataset contains a total of 18 874 proteins.

^b^Secreted: predicted by four predictors; HLS: highly likely secreted, predicted by three out of four predictors; LS: likely secreted, predicted by two out of four predictors; WLS: weakly likely secreted, predicted by one out of four predictors.

## Results

### Prediction accuracy evaluation

#### Mitochondrial proteins

The accuracy results are shown in Table 1a. When an individual tool was used, WoLF PSORT prediction showed a slightly lower sensitivity but a higher specificity than TargetP prediction. Thus, the MCC value was higher in the set predicted by WoLF PSORT (0.53) than the set predicted by TargetP (0.44). If only positives predicted by both tools were used, the specificity was slightly increased and the MCC value remains unchanged (0.53) compared with WoLF PSORT prediction. In contrast, including positives predicted by either tool decreased the MCC value to 0.45. Thus we assigned mitochondrial subcellular locations to entries only predicted to be mitochondrial proteins by both programs. As the specificity was high (up to 98.5%) when both tools were used, these predicted entries were reasonably reliable. However, the prediction sensitivity (42.5%) of the tools was low, i.e. more than half of proteins located in mitochondria remained to be predicted. Thus future efforts need to be made to improve prediction sensitivity for mitochondrial proteins.

#### Secreted proteins

Our previous evaluation showed that secreted prediction accuracy can be improved by removing transmembrane proteins, which can be predicted using TMHMM, and ER resident proteins, which can be predicted using PS-Scan (19). As we employed four tools—SignalP (version 4), TargetP, WoLF PSORT and Phobius—for predicting secreted proteins or secretory signal peptides, we had to determine which should be included in the secretome set. After removing transmembrane proteins and ER proteins, the protein set predicted either to contain a secretory signal peptide or to be secreted are divided into four categories: (i) Secreted: predicted by 4 predictors; (ii) Highly likely secreted (HLS): predicted by 3 out of 4 predictors; (iii) Likely secreted (LS): predicted by 2 out of 4 predictors; and (iv) Weakly likely secreted (WLS): predicted by 1 out of 4 predictors. The dataset consisted of 5724 curated secreted proteins as positives and 13 150 proteins located in other subcellular locations as negatives. The accuracy results are shown in Table 1b.

As expected, when only entries were predicted by all four tools to be positives as true positives, the prediction specificity was increased. However, the sensitivity was decreased. On the other hand, the prediction specificity was decreased but the sensitivity was increased when including all entries predicted by any of the four tools to be positives as true positives. Based on the MCC values, the most accurate prediction (0.89) for a secretome includes secreted entries predicted by at least three out of four predictors with a specificity of 96.0% and a sensitivity of 93.5% (Table 1b). Thus, we recommend including only curated secreted proteins and highly likely secreted proteins for estimating the secretome size. Though including the set of likely secreted proteins increased the coverage of a secretome, it increased more (272 entries) false positives than true (63 entries) positives. It should be noted that both entries predicted by 4 of 4 tools and 3 of 4 tools were assigned as the category of highly like secreted in the database, making them distinguishable from curated secreted entries.

#### Proteins in other subcellular locations

Proteins for the cytoplasm subset also include cytosol as these two terms are used interchangeably in the UniProtKB annotation. However, we noticed that the annotated cytoskeleton entries are also annotated as cytoplasm. In our evaluation, cytoskeleton proteins were not counted in the subset of cytoplasm. We would also like to point out that plasma membrane proteins were annotated as cell membrane in UniProtKB, thus cell membrane proteins were retrieved for evaluating the category of plasma membrane. The prediction accuracy results for proteins located in cytoplasm, cytoskeleton, ER, Golgi apparatus, lysosome, nucleus, peroxisome, plasma membrane and vacuole are shown in Table 1c.

The prediction accuracies for these subcellular locations vary significantly. Predictions of proteins located in nucleus and plasma membrane were relatively accurate with a MCC value of 0.78 and 0.72, respectively. Predictions for proteins located in cytoplasm, cytoskeleton, and ER were highly specific (specificity 93.4–99.7%) with a MCC value of 0.42–0.46. However, the sensitivities (27.6–55.6%) need to be improved for these subcellular locations. Predictions for proteins located in Golgi apparatus, lysosome, peroxisome were also highly specific (specificity > 99%) but with a very low sensitivity (0.5–4.5%). Human and animal vacuolar proteins could not be predicted by WoLF PSORT as there were no positive being predicted (Table 1c). It should be noted that the low MCC values for some of the subcellular locations were caused by low sensitivities, and in fact, the specificities were relatively high. Thus, there are a good number of proteins located in these subcellular locations not being predicted. However, if a protein is predicted to be located in such a location, the prediction is most likely reliable.

### Database statistics: subcellular proteome distribution in different species

The database contains curated and predicted subcellular location information of 4 080 818 metazoan proteins that were downloaded from UniProtKB. These proteins were generated from 185 256 metazoa species and subspecies with 121 of them having a complete proteome. Species specific proteins located at each subcellular location can be searched and downloaded from the database user interface. The distributions of subcellular proteomes in human and different animal species having a complete proteome are summarized in Table 2 and Supplementary Table S1. Table 2 includes the following subcellular locations: secreted proteins (3 subcategories), mitochondrial membrane and mitochondrial non-membrane, cytoplasm (cytosol), nuclear membrane and nuclear non-membrane, plasma membrane. The category of secreted proteins includes the following subcategories: curated secreted, highly likely secreted and likely secreted. Information on other subcellular protein locations including weakly likely secreted, cytoskeleton, ER (membrane or lumen), Golgi apparatus (membrane or lumen), lysosome, peroxisome, vacuole (membrane or non-membrane), other membrane, other curated locations and the information of species taxonomy can be found in Supplementary Table S1.

**Table 2.** Summary of proteins located in some major subcellular locations in human and different animal species

	Referenceproteome	Totalproteins	Curatedsecreted	Predicted		Mito			Nuc		Plasmamem		Secr(%)
				HLS	LS	mem	non-mem	Cyt	mem	non-mem		Secr	
Vertebrata (Actinopterygii)													
*Oryzias latipes*	24 633	26 060	144	1805	649	141	1185	7330	162	8629	3580	1949	7.5
*Xiphophorus maculatus*	20 451	20 527	92	1476	510	73	959	5288	92	7237	3052	1568	7.6
*Oreochromis niloticus*	26 753	27 551	122	2179	638	148	1051	6971	149	9638	4078	2301	8.4
*Gasterosteus aculeatus*	27 248	28 110	114	1813	618	106	1418	8080	142	9443	3877	1927	6.9
*Takifugu rubripes*	47 856	49 090	261	2655	1028	172	1645	12 630	323	17 843	8443	2916	5.9
Tetraodon nigroviridis*	23 073	49 327	194	2700	1236	182	2248	13 333	337	16 961	5944	2894	5.9
Danio rerio*	41 054	55 414	372	4635	1189	282	2319	14 909	267	19 521	6866	5007	9.0
Vertebrata (Amphibia)													
Xenopus tropicalis*	23 491	30 521	194	1926	656	169	1327	9674	168	10 086	4113	2120	6.9
*X. laevis*		16 011	269	1059	262	161	752	5124	109	5711	1636	1328	8.3
Vertebrata (Mammalia)													
Glires													
*Oryctolagus cuniculus*	21 150	22 788	334	1670	479	135	1222	5692	150	7241	3665	2004	8.8
*Heterocephalus glaber*	21 449	21 548	93	1266	513	90	1009	6343	103	6924	3266	1359	6.3
*Cavia porcellus*	19 911	20 378	236	1432	461	100	1016	5349	103	6410	3342	1668	8.2
*Cricetulus griseus*	23 884	24 442	109	1407	927	96	1170	7073	116	7114	2786	1516	6.2
Mus musculus*	43 539	74 158	1792	4350	1698	717	3443	20 456	714	23 226	9137	6142	8.3
Rattus norvegicus*	27 340	33 555	966	2211	637	476	1473	9153	411	10 094	5407	3177	9.5
*Spermophilus tridecemlineatus*	19 966	20 079	110	1437	429	83	937	5488	103	6603	3290	1547	7.7
Primates													
Macaca fascicularis *	17 396	28 955	233	1912	928	359	1976	7639	121	8186	2970	2145	7.4
M. mulatta*	35 536	69 567	407	4554	1694	653	3719	18 667	326	23 502	7295	4961	7.1
*Gorilla gorilla gorilla*	27 286	27 371	212	1994	676	218	1480	6701	151	9481	3358	2206	8.1
Homo sapiens*	68 049	13 5661	2020	6682	3480	3737	17 623	34 825	877	34 274	10 607	8702	6.4
Pan troglodytes*	20 126	33 326	296	2241	820	447	1825	7966	137	11 618	4190	2537	7.6
*Pongo abelii*	22 785	24 529	237	1818	580	229	1457	6452	168	8228	2879	2055	8.4
*Nomascus leucogenys*	19 734	19 837	141	1489	518	114	1143	5053	99	6893	2457	1630	8.2
Callithrix jacchus*	42 025	55 085	244	3776	1280	195	2867	15 064	308	20 159	6178	4020	7.3
*Otolemur garnettii*	19 930	20 156	99	1515	480	93	1022	5226	96	6801	3099	1614	8.0
Carnivora													
*Canis familiaris*	25 439	28 362	345	1813	595	385	1491	7040	170	9489	4047	2158	7.6
*Mustela putorius furo*		38 826	173	2017	984	137	2127	11 785	154	12 830	4073	2190	5.6
*Neovison vison*		16 237	18	750	356	66	839	5636	52	5233	1507	768	4.7
Ailuropoda melanoleuca*	21 136	35 743	247	2086	779	176	1746	9975	162	11 905	5133	2333	6.5
*Felis catus*	20 303	21 230	196	1406	483	108	1065	5831	107	6791	3091	1602	7.5
Cetartiodactyla													
*Bos mutus*		18 922	150	1377	453	123	931	4911	85	5854	3159	1527	8.1
Bos taurus*	23 842	31 780	880	2215	620	508	1491	8171	317	9598	4492	3095	9.7
Sus scrofa*	26 054	33 962	645	2534	779	411	1560	9038	166	9463	4787	3179	9.4
*Camelus ferus*		20 028	67	1084	636	99	1132	5715	147	6257	2588	1151	5.7
Chiroptera													
*Pteropus alecto*	19 520	19 548	97	1162	488	74	1160	5364	121	6774	2447	1259	6.4
*Myotis brandtii*		19 301	58	1032	432	90	938	5806	104	6427	2250	1090	5.6
*M. davidii*	15 446	15 466	67	916	345	60	782	4530	73	5194	1816	983	6.4
*M. lucifugus*	20 650	20 899	143	1738	431	100	1052	5716	96	6782	2855	1881	9.0
Other mammalia													
*Loxodonta africana*	25 615	25 832	132	1744	556	128	1119	6554	129	8459	4835	1876	7.3
*Equus caballus*	22 676	27 841	272	1659	514	284	1042	8886	133	8701	3825	1931	6.9
*Tupaia chinensis*	20 824	20 851	85	1275	527	64	1149	5699	125	6701	3114	1360	6.5
*Sarcophilus harrisii*	22 388	22 565	107	1490	553	110	867	6368	102	7495	3495	1597	7.1
*Monodelphis domestica*	22 240	22 794	108	1505	485	84	1103	6398	106	7252	3930	1613	7.1
*Ornithorhynchus anatinus*	23 552	23 763	113	1202	698	103	1111	7229	95	7184	3157	1315	5.5
Vertebrata (Testudines + Archosauria group)													
Anas platyrhynchos *	16 377	31 879	139	1360	893	123	1269	10 542	148	9829	3316	1499	4.7
*Meleagris gallopavo*	16 537	16 991	114	892	413	75	673	5622	83	5377	2073	1006	5.9
Gallus gallus*	17 623	23 800	440	1640	581	278	1231	6403	147	7077	3282	2080	8.7
*Ficedula albicollis*	15 922	16 148	64	985	390	57	778	4669	81	5208	2021	1049	6.5
*Taeniopygia guttata*	18 141	19 724	85	716	432	77	972	6749	72	6197	2211	801	4.1
*Chelonia mydas*		19 066	71	880	478	71	794	6031	97	6384	2194	951	5.0
*Pelodiscus sinensis*		20 784	126	1271	492	64	798	6724	92	6703	2683	1397	6.7
Other vertebrata													
*Petromyzon marinus*		13 160	54	522	255	66	669	3945	34	3797	1239	576	4.4
*Latimeria chalumnae*	23 429	23 513	75	1270	593	87	993	8117	116	7349	2905	1345	5.7
*Anolis carolinensis*	19 109	19 562	81	1238	510	288	936	5727	89	6435	2478	1319	6.7
*Invertebrate*													
Chordata (Tunicata)													
Oikopleura dioica*	17 050	29 057	15	1864	1177	116	1493	10 785	92	8060	2540	1879	6.5
*Ciona intestinalis*	17 308	18 639	28	1507	601	95	738	6231	47	5014	1812	1535	8.2
*C. savignyi*	20 004	20 117	45	822	359	52	697	7792	55	5685	2535	867	4.3
Ecdysozoa (Arachnida)													
*Tetranychus urticae*	18 082	18 243	12	1891	685	95	701	5986	59	3615	2219	1903	10.4
*Ixodes ricinus*		16 199	1	3554	819	136	848	3528	97	3649	1246	3555	21.9
*I. scapularis*	20 473	21 162	21	1943	762	103	1433	5706	95	5905	1681	1964	9.3
*Rhipicephalus pulchellus*		11 205	1	1620	459	60	962	2380	49	3424	1231	1621	14.5
Ecdysozoa (Insecta)													
*Drosophila mojavensis*	14 525	15 086	21	2049	347	95	832	4302	74	4241	1700	2070	13.7
*D. virilis*	14 456	14 928	34	1941	354	84	770	4308	57	4323	1709	1975	13.2
*D. erecta*		15 116	44	2220	362	71	873	3918	59	4366	1721	2264	15.0
*D. grimshawi*	14 754	14 798	31	1861	362	62	773	4336	79	4195	1698	1892	12.8
*D. ananassae*	14 968	15 298	28	2139	349	64	791	4243	67	4503	1793	2167	14.2
D. melanogaster*	20 120	39 951	254	4761	923	269	2127	10 613	191	12 101	4659	5015	12.6
*D. persimilis*	16 754	16 861	28	2106	420	77	930	4846	68	5076	1701	2134	12.7
*D. pseudoobscura* pseudoobscura		17 047	48	2316	416	77	939	4632	71	5126	1950	2364	13.9
*D. sechellia*	16 134	16 361	37	2250	410	71	936	4464	54	4765	1734	2287	14.0
*D. simulans*	15 354	19 057	57	2372	436	100	1028	5483	56	5374	2165	2429	12.7
*D. willistoni*	15 447	15 564	25	1875	355	77	815	4808	60	4434	1722	1900	12.2
*D. yakuba*		17 257	41	2521	392	77	1006	4752	59	5024	1798	2562	14.8
*Megaselia scalaris*	11 463	11 503	10	773	417	31	449	4947	24	2402	639	783	6.8
*Anopheles darlingi*	10 447	11 686	8	793	272	93	758	3687	54	3971	1162	801	6.9
A. gambiae*	13 072	19 384	50	2610	410	87	1104	6027	57	4834	1869	2660	13.7
*Aedes aegypti*	16 654	17 683	54	2367	469	182	961	5052	56	4873	2008	2421	13.7
*Culex quinquefasciatus*	18 703	19 062	25	2345	534	128	1104	5751	73	5501	1866	2370	12.4
*Dendroctonus ponderosae*		23 650	14	1992	549	106	1153	8928	96	6087	2502	2006	8.5
*Tribolium castaneum*	16 502	17 074	26	1717	423	66	846	5830	50	4196	2109	1743	10.2
*Apis mellifera*	10 910	12 299	65	757	267	81	390	4440	45	3293	1714	822	6.7
*Camponotus floridanus*	14 787	14 801	15	662	329	45	744	5533	65	4078	1346	677	4.6
*Acromyrmex echinatior*	13 962	13 970	17	592	327	36	847	5226	62	4219	1253	609	4.4
*Atta cephalotes*	18 079	18 113	16	753	597	99	1094	6579	75	4715	1559	769	4.2
*Solenopsis invicta*	14 193	14 359	26	636	437	100	748	5413	31	3508	1120	662	4.6
*Harpegnathos saltator*	15 029	15 042	17	739	329	46	696	5484	60	4223	1299	756	5.0
*Nasonia vitripennis*	17 040	17 289	14	1545	305	65	701	6951	55	4883	1423	1559	9.0
*Bombyx mori*	14 767	17 915	125	1773	379	108	806	6293	54	4580	1681	1898	10.6
*Danaus plexippus*	16 253	16 358	34	1486	441	95	808	5657	66	4528	1493	1520	9.3
*Pararge aegeria*		15 104	12	428	561	75	850	5983	14	3763	503	440	2.9
*Rhodnius prolixus*	15 180	16 639	44	1420	537	41	562	6782	62	3769	1473	1464	8.8
*Acyrthosiphon pisum*	35 809	35 211	24	1834	814	102	1736	15 209	66	8622	1736	1858	5.3
*Pediculus humanus *subsp. corporis		10 847	11	513	257	37	349	4294	40	3174	1193	524	4.8
Ecdysozoa (Nematoda)													
Ascaris suum*	9213	18 539	39	1223	577	107	1302	5894	75	4965	2437	1262	6.8
*Pristionchus pacificus*	29 079	29 319	14	3027	1038	75	1368	9699	94	7157	3263	3041	10.4
*Caenorhabditis brenneri*	29 982	30 712	21	3602	896	76	1134	10 314	88	7338	4255	3623	11.8
*C. briggsae*	21 751	21 914	30	2734	655	119	874	6435	106	5178	3540	2764	12.6
*C. elegans*	26 173	26 447	182	3573	856	163	1065	7156	107	6190	4401	3755	14.2
*C. japonica*	35 063	35 069	14	2665	998	95	2061	12 267	123	9112	3234	2679	7.6
*C. remanei*	31 252	32 133	21	3859	1117	84	1282	10 352	93	7199	4981	3880	12.1
*Haemonchus contortus*		18 580	3	2181	558	83	1016	5826	77	4679	2261	2184	11.8
Brugia malayi*	1643	11 561	10	668	347	37	579	4540	44	3139	908	678	5.9
*Loa loa*	15 319	15 356	11	784	588	46	750	5774	49	3749	1387	795	5.2
*Wuchereria bancrofti*	19 298	19 525	18	716	677	129	870	8254	39	4504	1205	734	3.8
*Trichinella spiralis*	16 041	16 278	17	935	770	73	980	5389	71	3433	1234	952	5.8
Ecdysozoa (Arthropoda)													
*Daphnia pulex*	30 137	30 988	22	2827	892	333	1432	11 433	88	8315	2130	2849	9.2
*Strigamia maritima*	14 972	15 011	19	1118	428	48	726	5331	68	3635	1835	1137	7.6
Lophotrochozoa													
*Helobdella robusta*	23 328	23 379	19	1170	671	59	924	9385	145	5866	2226	1189	5.1
*Capitella teleta*		31 207	22	2183	907	76	1263	10 827	106	7765	3917	2205	7.1
*Crassostrea gigas*	25 982	26 850	26	1904	633	85	814	10 178	140	7045	2912	1930	7.2
*Lottia gigantea*		23 721	34	1683	588	48	659	9382	76	5530	2734	1717	7.2
Platyhelminthes													
*Echinococcus granulosus*		11 124	0	614	375	381	656	2855	40	3518	1260	614	5.5
*E. multilocularis*		10 572	0	591	326	91	656	2878	48	3532	1239	591	5.6
*Clonorchis sinensis*	13 606	13 880	6	562	349	55	990	4294	89	5074	1234	568	4.1
*Schistosoma japonicum*		16 236	17	1767	607	70	853	6086	36	3511	1357	1784	11.0
*S. mansoni*	11 723	12 836	9	605	427	203	505	4491	60	3740	1242	614	4.8
Other Invertebrates													
*Amphimedon queenslandica*	29 741	29 816	6	1490	893	65	1246	11 722	73	7333	2685	1496	5.0
*Nematostella vectensis*	24 435	25 035	61	1135	586	58	1005	8651	72	6385	3293	1196	4.8
*Strongylocentrotus purpuratus*	28 567	29 560	46	2198	737	94	1101	9895	145	8580	4026	2244	7.6
*Trichoplax adhaerens*	11 520	11 590	7	482	213	36	489	5013	44	2502	1776	489	4.2
*Branchiostoma floridae*	28 544	29 237	37	2227	710	152	1146	8799	140	7800	3826	2264	7.7

*Notes*: Data of other protein subcellular locations are summarized in Supplementary Table 1. HLS: highly likely secreted; LS: likely secreted; Mito: mitochondrial; mem: membrane; non-mem: non-membrane; Cyt: cytoplasm (or cytosol); Nuc: nuclear; Secr: secretome. Species labeled with * has more (or less) 5000 protein entries than its reference proteome.

It should be noted that the distribution data of protein subcellular locations in Table 2 and Supplementary Table S1 were based on all available protein entries for each species in the database, which were different from a complete or reference proteome in some species. Several species had more redundant proteins in the dataset. For example, human reference proteome contained 68 049 proteins while a total of 135 661 human proteins were retrieved and used for analysis (Table 2). Thus, the proportions of each subcellular proteome might be slightly different for some species when a reference proteome was used. The two largest compartments having a large proportion of proteins were cytoplasm and nucleus (Table 2). The proteins located in cytoplasm, not including cytoskeleton proteins, accounted for 21–43% (average 31%), and the proteins located in nucleus accounted for 20–37% (average 30%) of total proteins in these species. Approximately 3–19% (average 12%) of total proteins are predicted to be plasma membrane proteins, and 3–9% of proteins (average 5.6%) are predicted to be located in mitochondria. We noticed that 15.7% of human proteins are located in mitochondria. This number is much higher than the proportions in other species. This might be due to relatively a large number (∼7000) of curated human mitochondrial proteins in the dataset. Also, the prediction sensitivity for mitochondrial proteins was relatively low (∼42.5%) (Table 1), thereby likely underestimating the proportions of mitochondrial proteins in animal species reported here.

Classical secreted proteins from a species, i.e. secretome, can be relatively accurately predicted. Combining curated secreted proteins and predicted highly likely secreted proteins (at least 3 positives out of 4 predictors) as a secretome, our method for a secretome prediction reached a MCC of 0.89 with 93.5% in sensitivity and 96.0% in specificity (Table 1). The proportions of secretomes vary from 2.9% to 21.9% with an average of 8.1% in animal species*. Pararge aegeria*, the Speckled Wood butterfly, had the smallest secretome of 440 proteins (2.9%), and *Homo sapiens* (human) has the largest secretome of 8702 proteins with 2020 proteins curated as secreted. However, human protein dataset contained a large proportion of redundant entries. After mapping to the human reference proteome, a total of 4969 secreted proteins (∼7.3%) were identified (see next section, Table 3). After excluding species having a large number (>5000 proteins) of duplicated protein entries (species labeled with * in Table 2) and using human secreted proteins mapped to human reference proteome, we plotted the secretome size and proteome size of remaining 103 species (Figure 1). Overall there is a good correlation between the proteome size (X) and the secretome size (Y) with a correlation coefficient of 0.658 (*Y* = 289.9 + 0.066X). However, clearly the secretome size is not only determined by its proteome size in a species. There are variations among different species. For example, secretomes in mammals had a range of 4.7–9.7% (average 7.3%), while the proportions of secretomes in insecta were more variable from 2.9 to 15% (average 9.8%), with *Drosophila* species had an average of 13.5% secretome (Table 2). We also noticed that among five species in *Caenorhabditis*, four exhibited a secretome accounting >11% of its proteome (Table 2). *Caenorhabditis* is a genus of nematodes that live in bacteria-rich environments like compost piles, decaying dead animals and rotting fruit. Their large secretomes may be related to their lifestyle for digesting complex biomolecules. Recently Suh and Hutter identified 3484 putative secreted proteins *C. elegans*, which were retrieved from WormBase (34). Interestingly, their retrieved numbers for potential secreted proteins and trasmembrane proteins (5458) in *C. elegans* closely coincide with our predictions (3755 secreted proteins and 5548 transmembrane proteins).

**Figrue 1.**
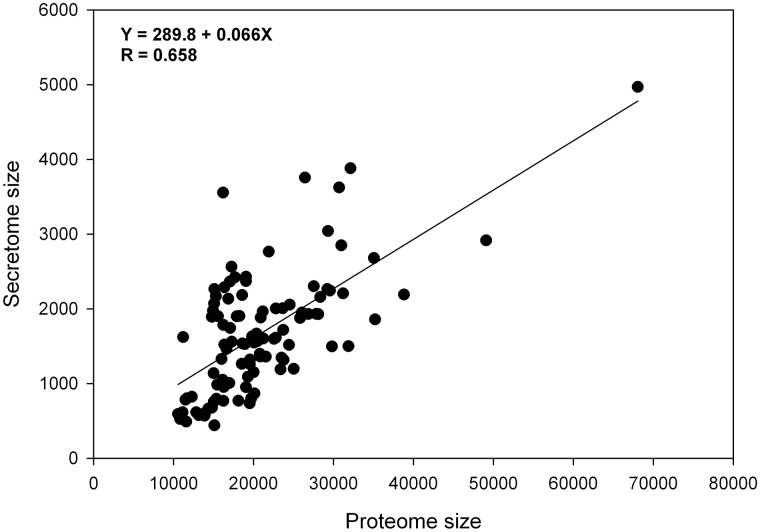
Relationship between the predicted secretome size and the proteome size in metazoa.

**Table 3.** The secretome size and the proportion of secretome relative to their reference proteomes in different primates

	Hsap	Cjar	Ggor	Mfas	Mmul	Nleu	Ogar	Ptro	Pabe
Secretome	4969	3204	2198	1460	2848	1617	1604	1852	1923
Secretome (%)	7.3	7.6	8.1	8.4	8.0	8.2	8.0	9.2	8.4

*Note*: The reference proteome size can be found in Table 2. *Hsap: Homo sapiens; Cjar: Callithrix jacchus; Ggor: Gorilla gorilla gorilla; Mfas: Macaca fascicularis; Mmul: Macaca lulatta; Nleu: Nomascus leucogenys; Ogar: Otolemur garnettii; Ptro: Pan troglodytes; Pabe: Pongo abelii*.

### Comparative analysis of secretomes in primates

Completely analysing the secretomes of all species mentioned above (Table 2) is beyond the scope of this work. Here we selected the secretomes of nine primates for comparative analysis (Table 3). As there are some redundant entries in the dataset, we mapped the identified secreted proteins to the reference or complete proteomes that are compiled by UniProtKB (http://www.uniprot.org/taxonomy/complete-proteomes). Among the nine primate species, the proportions of secretomes remained unchanged in three of them and others showed a slight increase, for example, the proportion of human secretome increased from 6.4% in the whole collection to 7.3% in the complete proteome set (Tables 2 and 3). Among the nine primate species, human has the largest proteome consisting of 68 049 proteins and the largest secretome size consisting of 4,969 proteins (Table 3). The large proteome size in human is mainly due to intensive collection of proteins generated by alternative splicing of protein coding genes (35, 36). We also noted that *Macaca mulatta* has a much larger, nearly doubled, proteome and secretome size than *M. fascicularis* has (Table 3). Whether such a large difference in these two closely related species is caused by the extensive genome segment duplications in *M. mulatta* (37) needs to be further examined.

To provide an overview of the functionalities of primate secreted proteins, we categorized the predicted secreted proteins into protein families using the rpsBLAST tool to search the Pfam database with a cutoff E-value of 1e−10. The secretomes of primates can be classified into a total of 841 unique protein families. The summary of the Pfam analysis with 28 families having 17 or more entries in a family in human is shown in Table 4. A complete list can be found in Supplementary Table S2. The top 10 highly encoded secreted protein families in primates were Trypsin, Immunoglobulin V-set domain, Serpin (serine protease inhibitor), Small cytokines (intecrine/chemokine), wnt family, von Willebrand factor type A domain, Immunoglobulin I-set domain, Fibrinogen beta and gamma chains, CUB domain and C1q domain. There are both variations in the Pfam categories and the number of entries in each Pfam among different primates. The significance of these secreted proteins in primate development and evolution certainly needs to be further investigated.

**Table 4.** Comparison of protein families in primate secretomes

Pfam ID	Pfam Name	*Hsap*	*Cjar*	*Ggor*	*Mfas*	*Mmul*	*Nleu*	*Ogar*	*Ptro*	*Pabe*	Pfam description
Total		2586	2222	1573	992	1907	1128	1187	1300	1349	
pfam00089	Trypsin	148	100	94	54	92	58	76	78	77	Trypsin
pfam07686	V-set	72	100	61	93	77	21	49	13	106	Immunoglobulin V-set domain
pfam00079	Serpin	60	30	23	22	25	16	20	20	23	Serpin (serine protease inhibitor)
pfam00048	IL8	42	34	35	28	38	34	23	34	33	Small cytokines (intecrine/chemokine)
pfam00110	wnt	42	36	25	16	26	21	19	22	20	wnt family
pfam00092	VWA	39	51	29	12	24	17	20	23	18	von Willebrand factor type A domain
pfam07679	I-set	37	28	16	13	21	14	9	22	12	Immunoglobulin I-set domain
pfam00147	Fibrinogen_C	32	37	25	14	24	21	19	19	22	Fibrinogen beta and gamma chains
pfam00431	CUB	32	23	12	4	20	6	8	9	9	CUB domain
pfam00386	C1q	30	39	24	12	22	18	27	25	17	C1q domain
pfam00019	TGF_beta	25	30	29	18	27	20	25	23	23	Transforming growth factor beta like domain
pfam00754	F5_F8_type_C	25	9	4	4	4	5	4	5	8	F5/8 type C domain
pfam01403	Sema	25	20	8	7	11	10	3	7	6	Sema domain
pfam00413	Peptidase_M10	24	17	21	14	18	15	13	15	12	Matrixin
pfam00059	Lectin_C	23	38	27	16	21	18	15	16	18	Lectin C-type domain
pfam05986	ADAM_spacer1	23	31	18	9	19	13	13	16	17	ADAM-TS Spacer 1
pfam00151	Lipase	22	10	10	7	8	9	7	7	7	Lipase
pfam00061	Lipocalin	19	25	22	8	14	6	18	10	8	Lipocalin/cytosolic fatty-acid binding
pfam00167	FGF	19	16	14	4	10	7	12	14	14	Fibroblast growth factor
pfam00193	Xlink	19	17	10	5	14	6	7	8	8	Extracellular link domain
pfam02931	Neur_chan_LBD	19	2	0	1	1	0	0	0	0	Neurotransmitter-gated ion-channel ligand
pfam03024	Folate_rec	19	6	4	3	3	3	4	4	5	Folate receptor family
pfam00530	SRCR	18	6	3	0	3	3	4	4	4	Scavenger receptor cysteine-rich domain
pfam00055	Laminin_N	17	26	14	3	22	9	10	11	9	Laminin N-terminal (Domain VI)
pfam00143	Interferon	17	11	14	8	16	11	10	13	13	Interferon alpha/beta domain
pfam00246	Peptidase_M14	17	18	14	8	15	12	9	14	12	Zinc carboxypeptidase
pfam07546	EMI	17	10	8	3	7	4	7	7	3	EMI domain
pfam13895	Ig_2	17	5	2	0	6	3	1	8	1	Immunoglobulin domain

*Note*: A complete list is shown as Supplementary Table 2. The species full names can be found in the note of Table 3.

**Figure 2.**
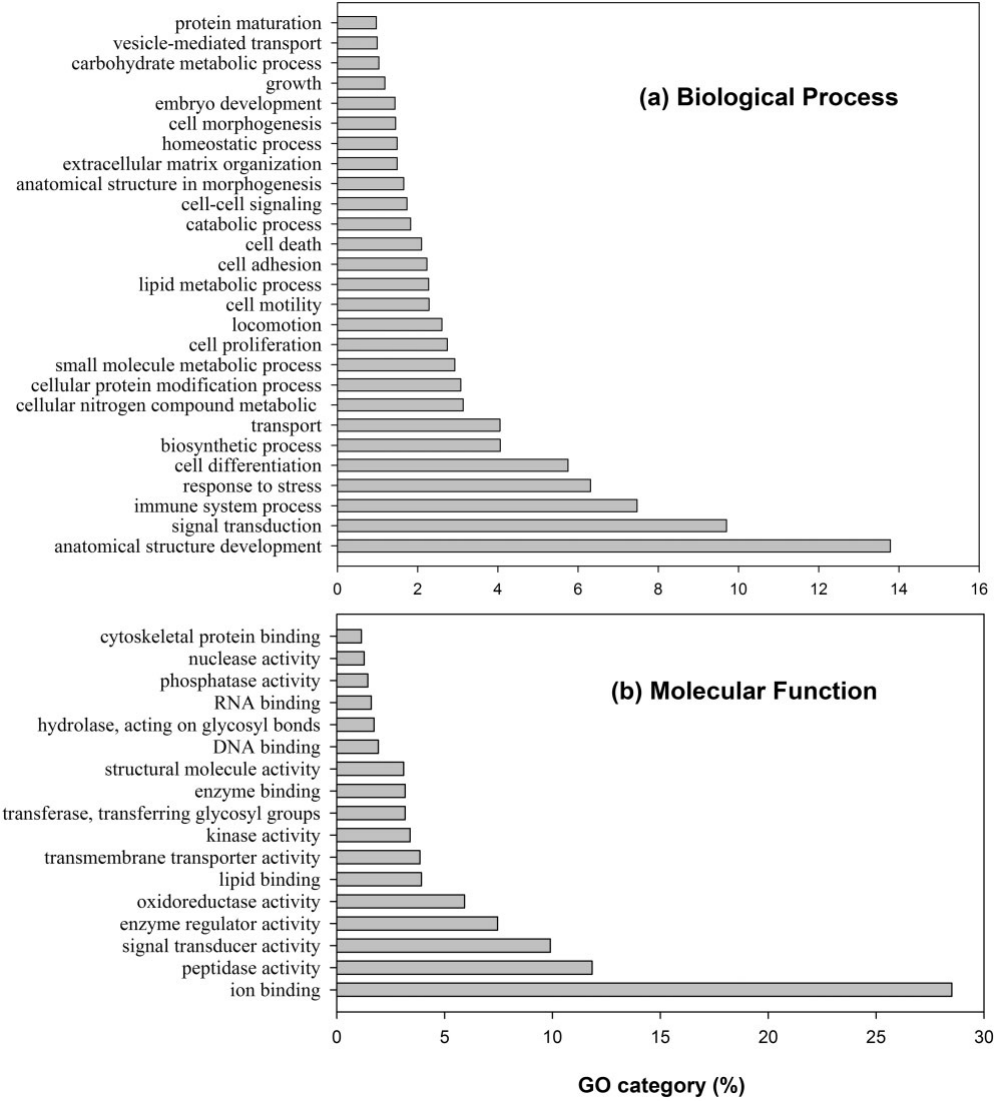
Gene Ontology classification of the human secreted protein distribution in (**a**) biological process and (**b**) molecular function ontology.

We further performed Gene Ontology (GO) analysis with the human secretome by searching the UniProtKB/Swiss-Prot dataset using BLASTP with a cutoff E-value of 1e−10. GO information was retrieved from UniProt ID mapping data (http://www.uniprot.org/downloads) and analysed using GO SlimViewer with generic GO terms (38). Among 4969 human secreted proteins, 4,512 entries had at least one GO mapping. As the proteins in the dataset are predicted to be secreted, thus, only GO biological process and molecular function classification is further analysed (Figure 2; Supplementary Table S3). Secreted proteins in humans are involved in 67 biological processes with a total of 25,887 GO IDs. The top five processes include anatomical structure development (13.8%), signal transduction (9.7%), immune system process (7.5%), response to stress (6.3%), and cell differentiation (5.8%) (Figure 2a). Molecular function analysis revealed human secreted proteins had 39 types of molecular functions with a total of 3,059 GO IDs. The top five main molecular functions include ion binding (28.5%), peptidase activity (11.8%), signal transducer activity (9.9%), enzyme regulator activity (7.5%) and oxidoreductase activity (5.9%) (Figure 2b). GO analysis and functional protein family domain analysis are consistent in showing these proteins play important roles in signal transduction, immune system, regulation of human structure development and many other biological processes.

## Discussion

The work described here represents our efforts to computationally predict the subcellular locations for all human and animal proteins, with a focus on secretomes. In addition, for the secretomes, we further classified them as curated, predicted to be highly likely secreted, likely secreted, and weakly likely secreted protein subsets. This refinement of classifications of secreted proteins and other subcellular locations is expected to greatly facilitate comparative analysis of subcellular proteomes in different species. Human secretome research is an active research subject due to its importance in human health and medicine, such as the human secretome atlas initiative with a goal for identifying potential biomarkers and therapeutic targets in the secretome that can be traced back in accessible human body fluids (12). For example, recently the human secreted enzyme Notum was found to inhibit the Wnt signaling pathway through removal of a lipid that is linked to the Wnt proteins and that is required for activation of Wnt receptor proteins (39, 40). Analysis of the secretome can yield valuable data leading to an understanding of the intricate interaction between different tissues as it relates to the coordination of physiology in multicellular organisms. An example is found in the interaction between muscles and bones (41). Many muscle specific growth factors, in the myosecretome, have been shown to have effects on bone repair and remodeling. Myostatin, a myocyte derived growth factor that inhibits muscle growth and thus acting as a break on uncontrolled growth, also has effects on suppression of bone marrow-derived stem cells and cartilage formation (41). In this study, we compared secretomes in different primates, and revealed that the highly enriched families including Trypsin, Immunoglobulin V-set domain, Serpin (serine protease inhibitor), Small cytokines (intecrine/chemokine) and wnt family, etc. Further we analysed the molecular functions and biological processes of the human secretome. Our analysis revealed the secreted proteins in humans play important roles in human structure development, immune systems, and response to stress, etc.

In this work, the secretome identification was limited to classical secreted proteins, i.e. signal peptide containing proteins, and curated secreted proteins that may include both classical and leadless-secreted proteins (LSP). SecretomeP was a tool implemented for predicting these LSPs in bacteria and mammals (http://www.cbs.dtu.dk/services/SecretomeP/). Because the accuracy of this tool for predicting animal LSPs is not evaluated, we did not include this tool in our data processing. Thus we would like to request the research community to submit metazoan protein subcellular locations, particularly LSPs, with experimental evidence traceable from literature to the database. The information provided in the database, the easy to download feature, and BLAST tool to allow users to search all protein data or the secretome data will provide useful supports to researcher working in these subjects. Researchers working with a new protein sequence can predict protein subcellular locations using the tools we have used in this work or other available tools that were summarized by Meinken and Min (32) and Caccia *et al.* (42).

The LOCATE database was developed for the human and mouse protein subcellular locations using multiple sources of information including literature data and computational prediction (17). However, the limit of the database was only for human and mouse proteins and the database has not been updated since 2009. Recently a new database named COMPARTMENTS was developed for seven model organisms including yeast, Arabidopsis, human, mouse, rat, fruit fly and *Caenorhabditis elegans* (http://compartments.jensenlab.org) (43). Our database contains protein data from all available metazoan species, with 121 species or subspecies having a complete proteome, including these model organisms. For plant and fungal protein data, we have specifically developed the plant secretome and subcellular proteome knowledgebase (PlantSecKB) (30) and the fungal secretome and subcellular proteome knowledgebase (FunSecKB and FunSecKB2) (3, 4). The COMPARTMENTS database was implemented by integrating information from UniProtKB, STRING, GO annotations from respective model organism databases, text mining, as well as prediction information using WoLF PSORT and YLoc-HighRes methods. In comparing with our database, both used the annotation information from UniProtKB and WoLF PSORT was the common tool used for prediction information. However, some other tools are used in our database development including TargetP, SignalP, Phobius, TMHMM and PS-Scan. In contrast, the COMPARTMENTS database used YLoc-HighRes method and also STRING, GO annotations. And also the COMPARTMENTS database has developed an automatically updated web resource to update from the major eukaryotic model organisms. Our database remained static for the predicted information and will be updated periodically for manually curated data based on the literature. Thus LOCATE, COMPARTMETNS and MetazSecKB may complement each other as each of them had specific features derived from different sources or prediction tools. Therefore, we recommend researchers to cross search these databases for proteins from model organisms. However, we noticed that these databases used different identifiers for protein entries, thus the data may not be compared directly. We anticipate the MetazSecKB, along with our published fungal secretome and subcellular proteome knowledgebase (FunSecKB2) (4) and the newly developed protist secretome and subcellular proteome knowledgebase (ProtSecKB) (http://proteomics.ysu.edu/secretomes/protist/index.php), will serve the community valuable resources for proteome-wide comparative analysis and for investigating protein–protein interactions of host and fungal or protist pathogens.

## Supplementary Data

Supplementary data are available at *Database* Online.

Supplementary Data
