# Supplementary material for: MetazSecKB: the human and animal secretome and subcellular proteome knowledgebase
Source: Database (Oxford). 2015 Aug 8;2015:bav077. doi: 10.1093/database/bav077 (PMC4529745; doi:10.1093/database/bav077)
Supplement: Supplementary Data [file supp_2015_bav077_index.html]

MetazSecKB: the human and animal secretome and subcellular proteome knowledgebase — Supplementary Data 

# MetazSecKB: the human and animal secretome and subcellular proteome knowledgebase

## Supplementary Data

files

- Supplementary Data - zip file
